# Mutations of the phenylalanine hydroxylase gene in Iranian patients with phenylketonuria

**DOI:** 10.1186/s40064-015-1309-8

**Published:** 2015-09-23

**Authors:** Alireza Biglari, Fatemeh Saffari, Zahra Rashvand, Safarali Alizadeh, Reza Najafipour, Mehdi Sahmani

**Affiliations:** Department of Molecular Medicine and Genetics, School of Medicine, Zanjan University of Medical Sciences, Zanjan, Iran; Department of Pediatrics, Qazvin University of Medical Sciences, Qazvin, Iran; Faculty of Medicine, The Cellular and Molecular Research Center, Qazvin University of Medical Sciences, Qazvin, Iran; Department of Clinical Biochemistry and Genetics, Faculty of Medicine, Cellular and Molecular Research Center, Qazvin University of Medical Sciences, Qazvin, Iran

**Keywords:** Phenylketonuria, PAH gene, Iranian population, Mutation detection

## Abstract

Phenylketonuria (PKU) is an autosomal recessive disease which results from mutations in the phenylalanine hydroxylase (PAH) gene. The aim of this study was the identification of sixteen different mutations in Iranian patients with hyperphenylalaninemia. The mutations were detected during the characterization of PAH genotypes of 39 PKU patients from Qazvin and Zanjan provinces of Iran. PAH mutations have been analyzed by PCR and direct sequencing of PCR products of the promoter region and all 13 exons of PAH gene, including the splicing sites. A mutation detection rate of 74.3 % was realized. Two mutations were found at high frequencies: R176X (10.25 %) and p.P281L (10.25 %). The frequencies of the other mutations were: IVS2+5G>A (2.56 %), IVS2+5G>C (2.56 %), p.L48S (2.56 %), p.R243Q (2.56 %), p.R252Q (5.12 %), p.R261Q (7.69 %), p.R261X (5.12 %), p.E280K (2.56 %), p.I283N (2.56 %), IVS9+5G>A (2.56 %), IVS9+1G>A (1.28 %), IVS11+1G>C (1.28 %), p.C357R (1.28 %), c.632delC (2.56 %). The present results confirm the high heterogeneity of the PAH locus and contribute to information about the distribution and frequency of PKU mutations in the Iranian population.

## Background

Deficiency of hepatic phenylalanine hydroxylase (PAH) [EC.1.14.16.1], which converts phenylalanine to tyrosine, is the major frequent cause of hyperphenylalaninemia (Guldberg et al. 1998). This enzyme defect, causes toxic accumulation of phenylalanine in the body fluids and damage to the nervous system that can result in growth failure, microcephaly, mental retardation and neurobehavioral abnormalities (Zhang et al. 2005). Phenylketonuria (PKU) is one of the most common inborn disease of amino acid metabolism, characterized by mutation of the PAH gene (Williams et al. 2008). According the levels of phenylalanine, they are four categories: mild hyperphenylalaninemia (HPA), mild PKU, moderate-PKU, and classic-PKU. Classical PKU is the most severe form of this disorder. A phenylalanine restricted diet, can be useful to prevent the neurotoxic complication of Phe and its metabolites (Olsson et al. 2007). The prevalence of PKU varies worldwide. In Caucasians, the prevalence is about 1/10,000 live births (Olsson et al. 2007), while that in Iranian population was 1/3627 (Koochmeshgi et al. 2002). In fact, the high rate of consanguineous marriages in Iran may be a contributing factor to the high incidence. The human PAH gene is located on chromosome 12q23.2 and is 90 kb in size with 13 exons and 12 introns (Santos et al. 2010). So far, several hundred different mutations in this gene have been identified in PKU patients and listed in the PAH mutation Analysis Consortium database (http://www.Pahdb.mcgill.ca). The most frequently occurring type of these mutations are missense mutations (Scriver 2007). The PAH gene mutations demonstrate considerable ethnic groups and geographic areas variation (Zschocke 2003). Previous studies have shown a correlation between PAH genotype and metabolic phenotype in PKU and have suggested the phenotypic relations of particular mutation combinations (Desviat et al. 1997; Kayaalp et al. 1997; Romano et al. 1996). Mutation analysis of a given population can be useful for the better understanding functional aspects of mutant protein and the relationship between genotype/phenotype.

## Objectives

The purpose of this study was to identify the molecular basis of PKU in Iranian Patients. In addition, we examined the variation in all 13 exons of the PAH gene in 39 patients, predominantly from Qazvin and Zanjan provinces of Iran.

## Patients and methods

Thirty- nine unrelated children with PAH deficiency were enrolled for this study after obtaining informed consent from the parents. A total of 39 patients, 24 cases were from the province of Qazvin and 15 cases from the Zanjan region. The PAH activity was measured by conventional biochemical methods. Most of the patients were identified when they showed mental retardation and few patients were identified during neonatal screening. The subjects were with ages ranging from 1 month to 10 years old. The clinical criteria were classical PKU with blood phenylalanine concentrations >20 mg/dl (>1200 µm/L) (Guttler 1980). The study was approved by the ethics committee of Qazvin University of medical sciences.

### DNA analysis

Genomic DNA was isolated from the leukocytes in blood samples using a DNA purification Qiagen kit (Valencia, CA, USA). All 13 PAH exons and their flanking intronic sequences were amplified by PCR using primers designed by primer 3 software. The primers sequences can be provided upon request. PCR reaction were performed on the Gene AMP PCR System Verity, (Foster City, CA, USA). The PCR condition were 95 °C for 3 min, 30 cycles of 95 °C for 30 s, 45–60 °C for 30 s, 72 °C for 30 s followed by 72 °C for 5 min. Samples were electrophoresed in 2 % agarose gel. The PCR products were sequenced by ABI prism 3130 genetic analyzer (Applied Biosystems, USA) and compared with the human genomic DNA sequence in GenBank to identify the mutations.

## Results

In this study, we detected causative mutations on 49 of the 78 mutant alleles (diagnostic efficiency 74.3 %) (Table 1). These included: eight missense mutations (50 %), five splice mutations (31 %), two nonsense mutations (12.5 %) and one deletion (6.25 %) (Table 1). Exon 7, 6, 2 and the flanking intronic regions include 85.5 % of the mutant alleles. The p.R176X and p.P281L mutations were the most frequent (10.5 %) followed by p.R261Q (7.69 %), p.R261X and p.R252Q (5.12 %), accounting for nearly 40 % of all mutations. Mutations p.R261X and p.R252Q were less frequent. All other mutations had frequencies less than 3 %. Among the 39 unrelated families studied, 20 (51.2 %) were homozygote, 6 (15.3 %) heterozygote and 2 (5.12 %) were compound heterozygote and 11 (28.2 %) were no PKU- causing mutations. The following polymorphisms were detected in the PAH gene: p.L385L, p.Q232Q and p.V245V with the frequency of 84, 51 and 17 % respectively were shown the highest prevalence among the other polymorphisms (Table 2). Genotypes of 39 PKU patients are shown in Table 3.

**Table 1:** Spectrum and frequency of PAH mutations identified in 39 patients

Systematic name (DNA level)	Trivial name (protein effect)	Location	Mutation type	Number of alleles	Frequency (%)
c.168+5G>A	IVS2+5G>A	Intron 2	Splicing	2	2.56
c.168+5G>C	IVS2+5G>C	Intron 2	Splicing	2	2.56
c.143T>C	p.L48S	Exon 2	Missense	2	2.56
c.526C>T	p.R176X	Exon 6	Nonsense	8	10.25
c.632delC	p.P211>Hfs	Exon 6	deletion	2	2.56
c.838G>A	p.E280K	Exon 7	Missense	2	2.56
c.782G>A	p.R261Q	Exon 7	Missense	6	7.69
c.842C>T	p.P281L	Exon 7	Missense	8	10.25
c.781C>T	p.R261X	Exon 7	Nonsense	4	5.12
c.755G>A	p.R252Q	Exon 7	Missense	4	5.12
c.728G>A	p.R243Q	Exon 7	Missense	2	2.56
c.848T>A	p.I283N	Exon 8	Missense	2	2.56
c.969+1G>A	IVS9+1G>A	Intron 9	Splicing	1	1.28
c.969+5G>A	IVS9 +5G>A	Intron 9	Splicing	2	2.56
c.1199+1G>C	IVS11+1G>C	Intron 11	Splicing	1	1.28
c.1069T>C	p.C357R	Exon 11	Missense	1	1.28
Total (number of alleles identified)				49	74.3

**Table 2:** PAH polymorphisms identified in 39 patients

Systematic name (DNA level)	Trivial name (protein effect)	Location	Number of alleles	Frequency (%)
c.696A>G	p.Q232Q	Exon 6	40	51.28
c.735G>A	p.V245V	Exon 7	14	17.9
c.912G>A	p.Q304Q	Exon 8	2	2.56
c.1155C>G	p.L385L	Exon 11	66	84.61
c.168+19T>C	IVS2+19T>C	Intron 2	5	6.4
c.-71A>C	5-UTR	5-UTR	4	5.1
c.843T>A	p.P281P	Exon 8	2	2.56
IVS3-22C>T	c.353-22C>T	Intron 3	2	2.56
Number of alleles identified			135

**Table 3:** Distributional genotypes in 39 PKU patients

Genotype	Polymorphism	Number of patients
u/u	c.168+19T>C, c.1155G>C, c.696A>G	1
c.838G>Ap.E280K/c.838G>Ap.E280K	c.735G>A, c.912G>A, c.1155C>G	1
u/u	c.1155C>G	1
c.782G>Ap.R261Q/c.782G>Ap.R261Q	c.1155C>G	1
u/u	c.735G>A, c.1155C>G	1
u/u	c.168+19T>C, c.1155G>C, c.696A>G	1
c.842C>T-p.P281L/c.842C>T-p.P281L	c.696A>G, c.1155C>G	2
u/u	c.168+19T>C, c.1155G>C, c.696A>G	1
c.781C>T-p.R261X/c.781C>T-p.R261X	c.1155C>G	1
c.755G>A-p.R252Q/U	c.696A>G, c.1155C>G	1
c.842C>Tp.P281L/c.842C>Tp.P281L	c.696A>G, c.1155C>G	1
u/u	c.696A>G, c.1155C>G	1
c.755G>A-p.R252Q/U	c.-71A>C	1
IVS9+1G>T/U	c.696A>G, c.1155C>G	1
C781C>T-p.R261X/C781C>T-p.R261X	c.696A>G, c.1155C>G	1
c.782G>A-p.R261Q/c.782G>A-p.R261Q	c.696A>G, c.1155C>G	2
c.755G>A-p.R252Q/c.755G>A-p.R252Q	c.696A>G, c.1155C>G, c.168+19T>C	1
c.526C>T-p.R176X/c.526C>T-p.R176X	c.-71A>C	1
c.526C>Tp.R176X/c.526C>Tp.R176X	c.1155C>G	1
c.143T>C, p.L48S/c.143T>C, p.L48S	c.1155C>G	1
u/u	c.1155C>G	1
IVS2+5G>A/IVS2+5G>A		1
c.848T>A-p.I283N/c.848T>A-p.I283N		1
u/u	c.168+19T>C, c.1155C>G	1
c.526C>T-p.R176X/c.526C>T-p.R176X	c.696A>G, c.1155C>G	2
c.842C>T-p.P281L/IVS11+1G>C	c.696A>G, c.1155C>G	1
c.842C>T-p.P281L/c.842C>T-p.P281L	c.696A>G, c.1155C>G	1
c.632delC p.P211>Hfs/c.632delC p.P211>Hfs	c.1155C>G, c.735 G>A	1
u/u	c.735G>A, c.1155C>G	1
c.728G>A-p.R243Q/U	c.735G>A, c.1155C>G	1
IVS9+5G>A/U	c.735G>A, c.1155C>G	1
IVS9+5G>A/U	c.696A>G, c.1155C>G	1
c.728G>A-p.R243Q/c.1069T>C-p.C357R	c735G>A	1
u/u	c.696A>G, c.1155C>G	1
IVS2+5G>C/IVS2+5G>C	c.696A>G, c.843T>A, c.1155C>G	1
u/u	c.735G>A, IVS3-22C>T	1

*U* unidentified

## Discussion

In this study, we identified mutations in the PAH gene and to evaluate the genetic heterogeneity of PKU disease in 39 unrelated Iranian patients who had been referred to Qazvin and Zanjan provinces. From this experiment, 28 of 39 PKU patients were found to contain the mutation. Our analysis of the homozygosity of the mutations were nearly similar to that observed in northwestern Iranian populations (Bonyadi et al. 2010). The majority of the recognized mutations are situated in the catalytic domains (143–410 amino acid), and some of them (p.P281L, R252W) are located in the cofactor binding regions. The most common mutation in our samples is P.P281L. This data agreement with what was found in other group Iran (Bonyadi et al. 2010; Hamzehloei et al. 2012). The P.P281L mutation in exon 7 with a relative frequency of 10.5 % is C → T substitution lead to conversation of Pro to Leu at codon 281 of PAH. The another major mutation in our study was p.R176X (10.25 %), which is similar to the data obtained in population of Khorasan Razavi origin (Hamzehloei et al. 2012).Previous study on the genotype/phenotype association demonstrated generally a positive correlation between R176X mutation and classic phenotype(Acosta et al. 2001; Bueno et al. 2013). Several studies reported that the IVS10-11G>A mutation, a splice mutation in the end of intron 10, observed with a high incidence among in the Mediterranean region, Brazil and some parts of Iran including: East Azarbaijan, Semnan, Khorasan Razavi, Hamedan (Dianzani et al. 1995; Kleiman et al. 1994; Rivera et al. 1998; Zare-Karizi et al. 2011), however this mutation was not found in the present study. The virtual absence of this mutation in our study may reflect the regional variability of populations. The second most frequent mutation identified in present study, R261Q (7.69 %) occurs on a CpG mutation hotspot on exon 7, leads to the conversion of Arg to Gln at codon 261 of PAH. This mutation is a common mutation in the Mediterranean and southern Europe but has a very low incidence in Spain (Couce et al. 2013; Loeber 2007; Perez et al. 1994; Rivera et al. 1998; Rivera et al. 2011). Furthermore, the frequency of R243Q mutation has been reported to be 18.2 % in Chinese and 12 % in Koreans, whiles in the present study the frequency of this mutation was found to be 2.5 % (Song et al. 2005). Most mutant PAH alleles probably with influencing on PAH gene transcription and translation can decrease the intracellular stability of protein and finally reduce enzyme function completely. In this study, we also analyzed the association between mutations and polymorphisms. The c.755G>A mutation located on the same allele with the c.168+19T>C polymorphism. Also, we observed association between the p.Q232Q polymorphism and c.842C>T, C781C>T, c.782G>A, c.755G>A, c.526C>T mutations occurred on the same allele in cis form, that particularly in the final case,similar associations have been reported in previous study (Hamzehloei et al. 2012). The majority of mutant alleles (73 %) were located on exon 7 and 6 that is in agreement with previous studies in Iranian populations (Hamzehloei et al. 2012; Zare-Karizi et al. 2011).In addition, the novel gene variant c.1069T>C (p.C357R) has been seen in Iranian population for the first time. Thereby to plan detection strategy; the samples will be screened first for mutations in these regions. If mutations were not identified, the other exons and their adjacent will be tested.

## Conclusion

Our results of Iranian individuals with PKU confirm a heterogeneous spectrum of mutations, displaying different ethnic and geographical origins. Moreover, our findings were slightly different from other ethnic groups. These findings can be useful to genotype/phenotype relationship in patients and provide future some ability to confirmatory diagnostic testing, prognosis and predict severity of PKU patients.
